# Machine learning-based prediction of fever among under-five children in Ethiopia: A national-level study

**DOI:** 10.1371/journal.pone.0354265

**Published:** 2026-07-28

**Authors:** Birhanu Betela Warssamo

**Affiliations:** 1 Department of Statistics, College of Science, Bahir Dar University, Bahir Dar, Ethiopia; 2 Department of Statistics, College of Natural and Computational Science, Hawassa University, Hawassa, Ethiopia; University of Uyo, NIGERIA

## Abstract

Fever remains a leading cause of morbidity and mortality among children under the age of five, particularly in low-resource settings like Ethiopia. Most existing studies in Ethiopia have relied predominantly on traditional statistical techniques, which may not fully capture complex patterns and nonlinear relationships within the data. To address this gap, the present study applies multiple machine learning algorithms to determine the most accurate model for predicting fever among under-five children in Ethiopia. This study utilized a total of 8,592 weighted child samples from the 2016 EDHS. After preprocessing, the dataset was randomly split into 70% training and 30% testing sets. Five machine learning algorithms; LR, RF, SVM, GNB, and DT were developed to predict fever. SMOTE was applied to address class imbalance in the training data. Model performance was assessed using accuracy, AUC, sensitivity, specificity, precision, F1-score, and balanced accuracy. Association rule mining via the Apriori algorithm was used to identify frequent patterns associated with fever. The overall prevalence of fever among under-five children was 14% (n = 1,197). Among the algorithms tested, the RF classifier achieved the best performance (accuracy: 92.2%, AUC: 0.958, sensitivity: 98.7%, specificity: 85.6%, F1-score: 0.8761). RF Gini importance and SHAP values ranked region, diarrhea & maternal factors as top predictors. Association rule mining revealed ten strong rules linking predictor variables with fever occurrence. The RF classifier demonstrated superior performance in predicting fever and identifying key features among under-five children. The results confirm that machine learning algorithms can serve as effective tools for accurately predicting childhood fever. These findings provide valuable insights for policymakers and public health stakeholders, supporting data-driven decision-making for targeted fever prevention strategies in Ethiopia.

## 1. Introduction

Fever is an elevation of body temperature above 38°C and is one of the most common clinical manifestations of infection and illness among children. It may result from viral, bacterial, or parasitic infections, such as malaria, pneumonia, and influenza, as well as inflammatory conditions or adverse reactions to medications, including antibiotics and vaccines. Common symptoms accompanying fever include chills, sweating, dehydration, and malaise [1–3].

Fever remains a major public health concern in Sub-Saharan Africa (SSA), particularly among children under five years of age [4]. It is a leading cause of childhood morbidity and mortality and is associated with malnutrition, impaired growth and development, and increased risk of death [5–8]. In low- and middle-income countries (LMICs), the burden of fever among children is particularly high, with an estimated two-week prevalence of 18.8% [9]. In Ethiopia, fever is also a common health problem among under-five children. According to the Ethiopian Demographic and Health Survey (EDHS), a substantial proportion of children experience fever in the two weeks preceding the survey, indicating a persistent public health challenge and highlighting the need for improved preventive and predictive strategies [10,11].

In Ethiopia, fever in under-five children is mainly associated with common infectious diseases such as malaria, acute respiratory infections, and diarrheal diseases, which remain leading causes of childhood illness and health service utilization. These conditions frequently present with fever as an early and important clinical symptom and contribute significantly to child health burden and healthcare demand in the country. Despite ongoing public health interventions, under-five fever remains a key concern in both rural and urban settings of Ethiopia, particularly among socioeconomically disadvantaged populations.

Health service institutions generate and manage vast amounts of data that can be leveraged to improve disease prediction and decision-making. To enhance diagnostic accuracy and support evidence-based healthcare, reliable predictive systems are increasingly needed [12]. In this context, machine learning (ML) has emerged as a powerful analytical approach for addressing complex health-related challenges [13]. ML combines principles from artificial intelligence and statistical learning and is particularly effective at identifying hidden patterns and nonlinear relationships within large and complex datasets [14].

Compared with traditional statistical approaches, machine learning (ML) algorithms have shown strong predictive performance in many classification problems and are increasingly being applied in medical and public health research [15–18]. However, their performance is not universally superior to traditional statistical models. The relative predictive accuracy of ML and conventional approaches depends on several factors, including the structure and quality of the data, the characteristics of the outcome variable, the complexity of underlying relationships, sample size, and the modeling context [19,20]. In situations involving complex nonlinear patterns and high-dimensional data, algorithms such as random forest (RF), support vector machine (SVM), and artificial neural networks (ANNs) may provide improved predictive performance [21]. Conversely, traditional statistical models such as logistic regression may perform equally well or better when relationships are relatively simple and model assumptions are adequately satisfied [15–18]. For example, machine learning algorithms have been successfully applied to predict childhood respiratory infections and other pediatric health outcomes in low- and middle-income countries. Recent studies in Ethiopia and Sub-Saharan Africa have demonstrated the utility of RF, SVM, and related algorithms for predicting acute respiratory infections among under-five children and identifying important risk factors [22–24].

Although previous studies have applied ML techniques to various childhood health outcomes, evidence specifically focused on fever among under-five children in Ethiopia remains limited. More importantly, existing studies have largely emphasized predictive accuracy and variable importance, with limited attention to how combinations and interactions of socio-demographic, environmental, and health-related factors jointly shape fever risk. As a result, there is still insufficient epidemiological understanding of distinct high-risk child profiles and how multiple determinants cluster to produce vulnerability to fever in the Ethiopian context.

Therefore, beyond model comparison, this study contributes new public health knowledge by identifying the joint effects and interaction patterns of key determinants of fever, and by uncovering high-risk subpopulations of under-five children through association rule mining. This approach provides deeper epidemiological insight into how fever risk is structured within the population, which can support more precise targeting of preventive interventions and more efficient allocation of child health resources in Ethiopia.

In response to this gap, and given the persistent burden of fever among under-five children in Ethiopia, this study utilizes nationally representative EDHS data and advanced ML algorithms to predict fever and identify its key determinants and risk patterns.

## 2. Methods

### 2.1 Data source

The data is utilized from the 2016 EDHS. The 2016 EDHS is the most recent available dataset and forms part of the global Demographic and Health Survey (DHS) program, which is conducted every five years. It is a nationally representative household survey designed to collect comprehensive information on population and health indicators, with a focus on improving maternal and child health in Ethiopia [11]. The survey employed a two-stage cluster sampling design with urban–rural stratification across regions, resulting in 21 sampling strata and a total of 645 enumeration areas (clusters) 202 from urban and 443 from rural areas. In total, 15,683 households were included in the final sample, comprising 5,348 urban and 10,335 rural households. For this study, the analysis was based on a weighted sample of 8,592 children aged 0–59 months. Although the EDHS 2016 was conducted several years ago, it provides the comprehensive set of child, maternal, household, and environmental variables required for the present analysis. In contrast, the 2019 Mini-EDHS collected a more limited set of indicators and did not include several key variables used in this study.

### 2.2. Study design and setting

A cross-sectional study design was employed to analyze data from under-five children in Ethiopia, using the 2016 EDHS, which was conducted between January 18 and June 27, 2016.

### 2.3.Study variables

The outcome of interest in this study was fever among under-five children. In the EDHS 2016, fever was assessed based on the mother’s or caregiver’s report of whether the child had experienced fever during the two weeks preceding the survey. The outcome variable was coded as binary, where 1 indicates the presence of fever and 0 indicates the absence of fever. A range of covariates were selected as potential risk factors for childhood fever in Ethiopia. These variables were extracted from the EDHS dataset based on evidence and guidance from previous studies.

### 2.4. Data preprocessing and analytic strategies

**Fig 1:**
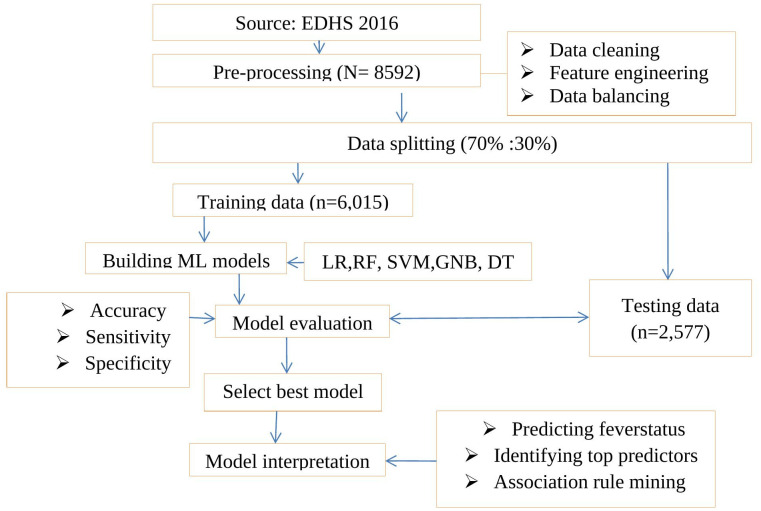
Overview of the study workflowworkflow.

The initial step in machine learning is data preprocessing, which entails transforming and encoding data into a format that can be interpreted by computers. This step plays a crucial role in enhancing the predictive performance of ML models [25]. Key data preparation techniques include data cleaning, feature engineering and splitting the data into training and testing sets [26]. The overall workflow of the study is summarized in Fig 1.

### 2.5.Data cleaning

Data cleaning was the initial and essential step conducted after retrieving the dataset. This process involved detecting **and removing outli**ers, addressing missing values, and managing class imbalance in the outcome variable [27]. To handle missing values, we employed the KNN imputation method [28]. KNN imputation was chosen due to its ability to retain the entire dataset, manage both numerical and categorical variables, handle outliers effectively, adapt to new data, and minimize bias across a broad range of values [29]. The proportion of missing values was range from 5% to 15% across variables. Variables with minimal missingness (<5%) were unlikely to impact results, while variables with higher missingness were carefully checked to ensure imputation did not introduce bias. Additionally, we assessed multicollinearity among the independent variables by examining the Variance Inflation Factor (VIF) [30].

### 2.6. Data splitting

To train and validate the model, we employed a 70/30 data split, where 70% of the samples were allocated for training and the remaining 30% were reserved for testing the model’s performance. In addition, tenfold cross-validation was applied during the training phase to enhance model reliability and reduce over fitting [31].

### 2.7. Imbalanced data handling

ML models trained on imbalanced datasets often become biased toward the majority class, resulting in poor prediction performance for the minority class. This imbalance can lead to reduced overall accuracy and biased estimates when classifying rare events or minority instances [32]. To address this challenge, it is important to apply data balancing techniques. In this study, we employed the Synthetic Minority Over-sampling Technique (SMOTE) to balance the class distribution and improve the model’s ability to detect cases in the minority class [33].

### 2.8. Model selection

After splitting the dataset into training and testing sets, suitable models were selected for training. Given that the outcome variable was categorical, the analysis was framed as a classification task, requiring the application of appropriate classification algorithms. Specifically, the dataset represented a binary classification problem, as the outcome variable, fever, was classified into two mutually exclusive categories: presence or absence of fever. To conduct the analysis, we employed five machine learning algorithms: LR, RF, SVM, Gaussian Naïve Bayes (GNB), and Decision Tree (DT). These classifiers were used to evaluate the predictive performance of ML models and to identify the most influential predictors of childhood morbidity, with a focus on fever.

These algorithms were selected based on their successful application in previous health-related ML studies, their suitability for binary classification problems, and their complementary characteristics in terms of interpretability, nonlinear pattern recognition, and computational efficiency, enabling a comprehensive comparison of predictive performance.

#### 2.8.1. Logistic regression.

LR is a supervised machine learning algorithm commonly used to address classification problems. It is a parametric method that assumes the target variable follows a Bernoulli distribution and that the observations are independent [34].

#### 2.8.2. Decision tree.

DT is a non-parametric classification technique that partitions a dataset based on the predictive structure of the problem. It generates a classification tree when applied to categorical outcomes. DTs are known for their high interpretability, ability to capture nonlinear relationships, and their flexibility in handling both categorical and numerical variables. They are also relatively robust to outliers and noisy data, can manage missing values using surrogate splits or imputation techniques, and are capable of efficiently processing large datasets [35].

#### 2.8.3. Random forest.

RF is a supervised ensemble learning algorithm that operates by constructing multiple decision trees during the training phase [36]. It repeatedly samples the training dataset and, for each tree, selects a random subset of predictor variables to generate a classification or regression tree. Once a large number of trees are built, the model aggregates their outputs using majority voting for classification or averaging for regression to make final predictions. RF is known for its robustness, flexibility, and speed, making it suitable for both classification and regression tasks. It effectively reduces over fitting compared to individual DT and provides measures of variable importance, helping to identify the most influential predictors in the dataset.

#### 2.8.4. Support vector machine.

SVM is a supervised learning algorithm used for classification, regression, and outlier detection [37**]**. SVM is particularly effective in handling high-dimensional datasets, is robust to outliers, and performs well when the data are well-separated. It works by finding the optimal hyper plane that maximally separates classes in the feature space, and can handle non-linear relationships through the use of kernel functions.

#### 2.8.5. Gaussian naïve bayes.

NB is a family of machine learning algorithms based on Bayes’ Theorem, built upon two key assumptions: (1) all features are independent of one another, and (2) each feature contributes equally to the prediction of the outcome [38]. A commonly used variant, Gaussian Naïve Bayes (GNB), assumes that continuous features follow a normal (Gaussian) distribution. GNB is particularly well-suited for problems where efficiency, simplicity, and the ability to handle continuous variables are important. It performs well with small training datasets, is effective in text classification, and excels in scenarios where the assumption of feature independence is reasonably satisfied. However, the performance of GNB can degrade when its assumptions are strongly violated. It may struggle with datasets in which features are highly correlated or when the decision boundary is complex and non-linear, limiting its ability to accurately classify such data.

### 2.9. Machine learning approach

In accordance with standard ML practices, the dataset was divided into training and testing subsets to enable model learning, training of classification algorithms, and pattern identification within the data. Once trained, the algorithms were applied to the test set, and their predictive performance was evaluated. Since the outcome variable represented a binary classification problem related to childhood morbidity, five machine learning algorithms were employed. The selection of these algorithms was informed by prior studies utilizing machine learning on EDHS data, as well as the nature of the problem and characteristics of the dataset.

Each selected classifier was trained on both balanced and unbalanced data, and the model demonstrating the best predictive performance was ultimately chosen. This optimal model was then trained on the balanced training dataset and used to make final predictions on the unseen test data.

Model performance was assessed using the confusion matrix and the receiver operating characteristic (ROC) curve. Evaluation metrics included accuracy, recall (sensitivity), specificity, F1 score, and area under the ROC curve (AUC). Among these, AUC was emphasized as the primary performance indicator, offering a comprehensive measure of classification performance across various thresholds. The confusion matrix provided detailed insights into model predictions by quantifying True Positives (TP), True Negatives (TN), False Positives (FP), and False Negatives (FN) [39].

All models were trained using 10-fold cross-validation, which involves partitioning the training data into ten equal subsets. The model was iteratively trained and validated ten times, each time using a different subset as the validation set and the remaining as the training set.

### 2.10. Algorithm evaluation

To assess the classification performance of the algorithms, a confusion matrix also known as an error matrix was utilized. In the context of binary classification, the confusion matrix is a 2 × 2 table that displays the counts of TN, FN, TP, and FP based on the predicted and actual class labels. This matrix provides a comprehensive summary of the model’s performance and enables the calculation of key evaluation metrics such as accuracy, sensitivity (recall), and specificity [40].

#### 2.10.1. Accuracy.

Accuracy is a fundamental metric for evaluating the performance of any predictive algorithm. It represents the proportion of correct predictions made by the model relative to the total number of instances evaluated. In this study, we reported the highest accuracies achieved by various machine learning algorithms after implementing feature selection and applying k-fold cross-validation techniques to ensure robustness and generalizability of the models.

$\text{Accuracy}=\frac{\text{TP}+\text{TN}}{\text{TP}+\text{FN}+\text{FP}+\text{TN}}$

#### 2.10.2. Sensitivity.

Sensitivity, also referred to as recall, measures the proportion of actual positive cases that are correctly identified by the model (i.e., true positives). It reflects the model’s ability to detect positive instances accurately. Conversely, the false negatives represent the proportion of actual positive cases that are incorrectly classified as negative. This misclassification can also be expressed as the false negative rate, which is the complement of sensitivity.

$\text{Sensitivity}=\frac{\text{TP}}{\text{TP}+\text{FN}}$

#### 2.10.3. Specificity.

Specificity measures the proportion of actual negative cases that are correctly identified as negative by the model (i.e., true negatives). It reflects the model’s ability to correctly exclude negative instances. Conversely, some actual negative cases may be incorrectly classified as positive, which are referred to as false positives. The rate of these misclassifications is known as the false positive rate, the complement of specificity.

$\text{Specificity}=\frac{\text{TN}}{\text{TN}+\text{FN}}$

### 2.11.Hyperparameter tuning and model optimization

To enhance model performance and ensure reproducibility, hyperparameter tuning was performed for all machine learning algorithms using 10-fold cross-validation implemented in the caret package in R software 4.3.1 for Windows (R Development Core Team). For the RF model, the number of variables randomly sampled at each split (mtry) was tuned over the grid {2, 4, 6}, while the number of trees (ntree) was set to 1000 to ensure model stability. For the SVM with a radial basis function (RBF) kernel, the cost parameter (C) and kernel width (σ) were optimized over the grid {C = 0.1, 1, 10} and {σ = 0.01, 0.05}. The DT model was tuned by varying the complexity parameter (cp) in the range of 0.001 to 0.05 to control tree pruning and prevent over fitting. For the NB classifier, the laplace smoothing parameter (fL), kernel density option (use kernel), and bandwidth adjustment (adjust) were tuned using the grid {fL = 0 or 1, usekernel = TRUE/FALSE, adjust = 1 or 2}. LR does not require hyperparameter tuning because it is estimated directly through maximum likelihood. For each algorithm, the optimal hyperparameter combination was selected based on the highest cross-validated accuracy. The final models were then retrained on the full training dataset using the selected hyperparameters and evaluated on the testing dataset.

### 2.12. Variable selection and encoding

Independent variables were selected based on theoretical relevance, prior empirical evidence, and significance in the bivariate analysis. Variables associated with fever at p < 0.25 in the bivariate analysis were included as candidate predictors in the multivariable machine learning models. Nominal categorical variables (e.g., region) were transformed using one-hot (dummy) encoding. For LR, one category from each variable was omitted and treated as the reference group. Ordinal variables were encoded according to their natural order.

### 2.13. Association rule mining

Association rule mining was performed using the Apriori algorithm implemented in the R package *arules* to identify frequent patterns of risk factors co-occurring with childhood fever. All predictor variables retained in the final machine learning models were eligible for inclusion. Continuous and ordinal variables were categorized based on standard DHS coding procedures. Each child’s categorized variables were converted into a binary transactional matrix, with the consequent restricted to fever (yes), ensuring that only rules predicting fever occurrence were generated.

A minimum support threshold of 0.05 and a minimum confidence threshold of 60% were selected a priori, based on prior literature and preliminary evaluation to balance rule frequency and relevance given the 14% prevalence of fever. To assess robustness, a sensitivity analysis was conducted by varying support (0.03–0.07) and confidence (60% – 90%). The top 10 rules were retained for interpretation based on a composite ranking of lift, support, and confidence, prioritizing rules with lift greater than 1 and epidemiological plausibility.

### 2.14. Ethics approval and consent to participate

This study is based on secondary data obtained from the 2016 EDHS, which is publicly available through the DHS Program website (https://dhsprogram.com). Ethical clearance for the original data collection was provided by the Ethiopian Public Health Institute (EPHI), the National Research Ethics Review Committee (NRERC), and ICF International’s Institutional Review Board. Before using the dataset, permission was obtained from the DHS Program by submitting a formal request and describing the purpose of the study. The dataset used was completely anonymized and did not contain any personally identifiable information.

## 3. Results

### 3.1. Study participant characteristics

In Table 1, a total of 8,592 weighted under-five children were included in the final analysis. Among them, 1,197 (14%) were found to be fever positive, with a 95% confidence interval ranging from 11.12% to 15.59%. Among children without diarrhea, the majority 6,859 (89.9%) did not experience fever, while 767 (10.1%) did. In contrast, among those with diarrhea, only 536 (55.5%) were free from fever, whereas a significantly higher proportion 430 (44.5%) experienced both conditions. The prevalence of fever also showed a slight upward trend with increasing maternal BMI. Specifically, among children whose mothers were underweight (BMI < 18.5), 13.3% had fever and 86.7% did not. This increased to 14.8% among children of mothers with a normal BMI (18.5–24.9), and to 15.2% among those whose mothers were overweight or obese (BMI ≥ 25). Among children reported as small at birth, 16.8% experienced fever, while 83.2% did not. In comparison, 11.9% of children with average birth size and 14.1% of those reported as large at birth had fever. Fever prevalence varied notably by region. The highest prevalence was observed in Tigray, where 23.7% of children were reported to have fever, followed by Afar (16.6%), Addis Ababa (14.9%), Oromia (14.7%), and the Southern Nations, Nationalities, and Peoples’ Region (SNNPR) (14.5%). In contrast, the lowest prevalence was recorded in Benishangul (7.6%) and Somali (9.0%). Regions such as Amhara, Harari, Gambela, and Dire Dawa showed moderate fever prevalence, ranging between 10.6% and 15.4%.

**Table 1:** Descriptive summary of socio-demographic variables for under-five children in Ethiopia, 2016 EDHS.

Variable	Categories	Fever prevalence		Chi-square (P- value)
		No n (%)	Yes n (%)	
Region	Tigray	672(76.3)	209(23.7)	134 (< 0.001)
	Afar	679(83.4)	135(16.6)	
	Amhara	752(87.6)	106(12.4)	
	Oromia	1134(85.3)	195 (14.7)	
	Somali	1045(91.0)	103(9.0)	
	Benishangul	652(92.4)	54(7.6)	
	SNNPR	901(85.5)	153(14.5)	
	Gambela	463(84.6)	84(15.4)	
	Harari	406(89.4)	48(10.6)	
	Addis Adaba	330(85.1)	58(14.9)	
	Dire Dawa	361(87.4)	52(12.6)	
Diarrhea	No	6859(89.9)	767(10.1)	570 (< 0.001)
	Yes	536(55.5)	430(44.5)	
Preceding birth interval	Less than 24	1637(87.2)	241(12.8)	24 (0.002)
	25-48	3853(86.3)	613(13.7)	
	49 and above	1905(84.7)	343(15.3)	
Succeeding birth interval	Less than 24	968(90.6)	100(9.4)	142 (< 0.001)
	25-48	3839(85.9)	628(14.1)	
	49 and above	2588(84.7)	469(15.3)	
Body mass index	Less than 18.5	4870(86.7)	747(13.3)	18 (0.018)
	18.5-24.9	443(85.2)	77(14.8)	
	>=25	2082(84.8)	373(15.2)	
Frequency of antenatal care visit	0-4	2057(83.7)	401(16.3)	150 (< 0.001)
	5-8	4717(87.7)	664(12.3)	
	9 and more	621(82.5)	132(17.5)	
Respondent employed	Occasional	325(78.1)	91(21.9)	152 (< 0.001)
	Seasonal	4779(86.0)	777(14.0)	
	All year	2291(87.4)	329(12.6)	
Sex of child	Male	3747(86.0)	612(14.0)	120 (< 0.001)
	Female	3648(86.2)	585(13.8)	
Households/ father age group	24 and younger	1709(83.4)	340(16.6)	25 (0.002)
	25-34	3927(86.8)	595(13.2)	
	35-44	1619(86.8)	246(13.2)	
	45 and older	140(89.7)	16(10.3)	
Number of child under 5 in household	Below 1	2482(83.2)	501(16.8)	142 (0.002)
	2-3	4707(87.8)	657(12.2)	
	4 and above	206(84.1)	39(15.9)	
Time to get water resource in min	Below 5	511(85.9)	84(14.1)	35 (0.023)
	6-16	1206(86.9)	182(13.1)	
	16-30	1836(87.0)	274(13.0)	
	30 and above	3842(85.4)	657(14.6)	
Age of mother at first birth	Under 15	986(86.1)	159(13.9)	147 (0.003)
	15-19	3579(85.6)	600(14.4)	
	20-24	2209(86.4)	349(13.6)	
	25 and older	621(87.5)	89(12.5)	
Marriage to first birth interval in months	Less than 12	1993(86.5)	310(13.5)	99 (0.012)
	12-24	2416(86.4)	379(13.6)	
	25 and more	2986(85.5)	508(14.5)	
Birth order of child	First order	1433(84.7)	259(15.3)	56 (0.011)
	Second order	1265(86.3)	200(13.7)	
	Third and above	4697(86.4)	738(13.6)	
Source of drinking water	Piped water	5656(86.2)	903(13.8)	76 (0.005)
	Public tap	1160(85.7)	193(14.3)	
	Protected spring	579(85.1)	101(14.9)	
Type of toilet facility	Flush toilet	288(85.2)	50(14.8)	256 (<0.001)
	Latrine	3838(86.6)	592(13.4)	
	No facility	3269(85.5)	555(14.5)	
Relation to household head	Head	1248(87.0)	187(13.0)	123 (0.002)
	Wife	5561(86.4)	873(13.6)	
	Other	586(81.1)	137(18.9)	
Wealth index household	Poor	4003(86.2)	641(13.8)	412(<0.001)
	Middle	1068(87.3)	156(12.7)	
	Rich	2324(85.3)	400(14.7)	
When child put to breast	After an hour	6262(86.7)	960(13.3)	121 (0.004)
	Within the first hour	393(88.9)	49(11.1)	
	Immediately	740(79.7)	188(20.3)	
Size of child at birth	Small	2011(83.2)	406(16.8)	96 (0.015)
	Average	3186(88.1)	430(11.9)	
	Large	2198(85.9)	361(14.1)	

### 3.2. Machine learning analysis

In this study, we applied five supervised machine learning algorithms LR, RF, SVM, NB, and DT to predict the occurrence of fever among under-five children in Ethiopia using the 2016 EDHS dataset. The data were preprocessed and balanced using the SMOTE technique to address class imbalance.

#### 3.2.1. Data balancing.

**Fig 2:**
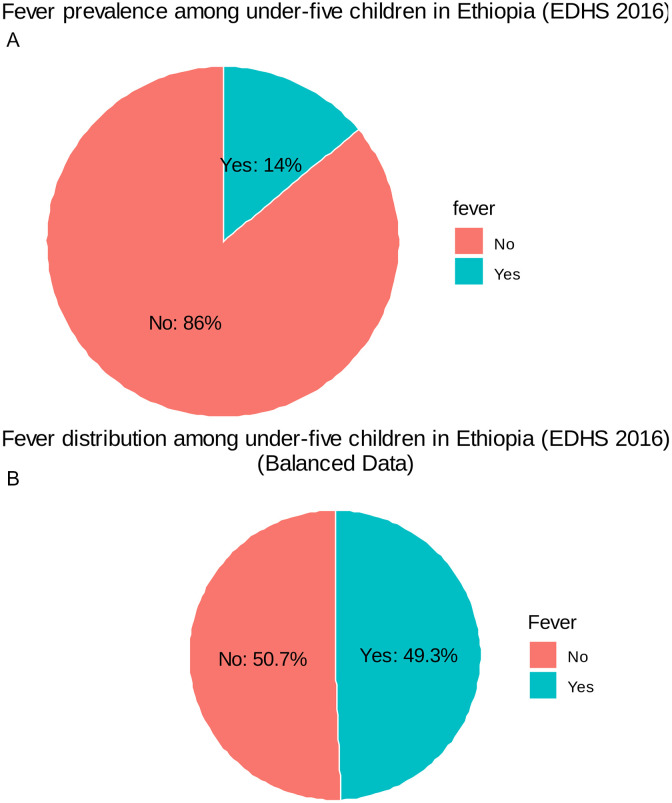
Prevalence of fever among under-five children in Ethiopia with unbalanced and balanced data (EDHS 2016).

Fig 2 presents the class distribution before and after applying SMOTE. To avoid data leakage and ensure unbiased performance estimation, SMOTE was implemented within each fold of the 10-fold cross-validation procedure, applied only to the training portion of each fold, while the validation fold remained untouched. Prior to balancing, 5,177 children (86.1%) did not have fever, while only 1,197 children (14%) had fever. After applying SMOTE, the dataset comprised 5,177 children without fever (50.7%) and 5,028 children with fever (49.3%), indicating a well-balanced distribution.

#### 3.2.2. Model performance evaluation.

**Fig 3:**
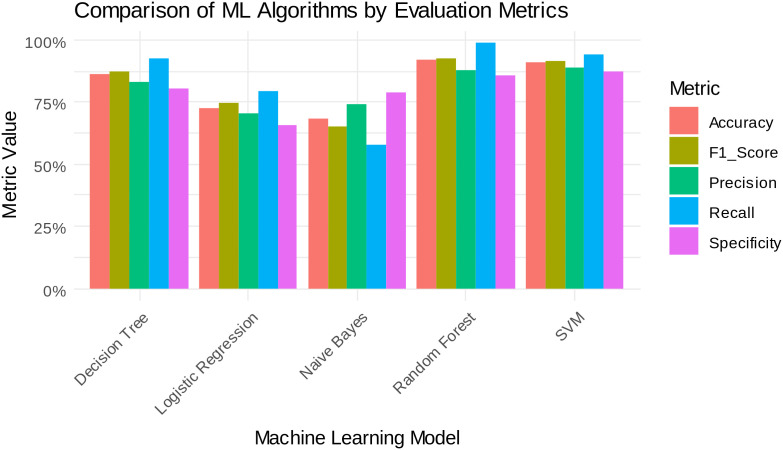
Comparison of model performance metrics after data balancing using SMOTE.

Table 2 presents the performance metrics of five machine learning models. A visual comparison of these performance metrics is presented in Fig 3. Among them, the RF model outperformed all others, achieving the highest accuracy 92.2% indicating excellent predictive performance. Very high sensitivity (recall) of 98.7%, indicating that it correctly identified nearly all fever cases, strong specificity at 85.6% reflecting a high capacity to correctly detect non-fever cases, and a Kappa statistic of 0.8442 indicates strong agreement between predicted and actual classifications well above what would be expected by chance, positive predictive value (precision) of 87.6%, and the negative predictive value of 92.8%, further confirming the model’s reliable predictions, F1-score of 0.8761 reflecting an optimal balance between precision and recall. The balanced accuracy, which averages sensitivity and specificity, was 92.2%, reaffirming the model’s consistent performance in identifying both fever and non-fever cases.

**Table 2:** Predictive performance summary of the five machine learning algorithms.

Metric	Accuracy Mean ± SD	Sensitivity (Recall) Mean ± SD	Specificity Mean ± SD	KappaMean ± SD	Precision (PPV) Mean ± SD	F1-scoreMean ± SD	NPV Mean ± SD	Balanced Accuracy Mean ± SD
Logistic Regression	0.7249 ± 0.02	0.7933 ± 0.03	0.6545 ± 0.02	0.4487 ± 0.03	0.7028 ± 0.02	0.7446 ± 0.03	0.7546 ± 0.02	0.7239 ± 0.02
Random Forest	0.9222 ± 0.01	0.9865 ± 0.01	0.8561 ± 0.04	0.8442 ± 0.02	0.8759 ± 0.03	0.9278 ± 0.02	0.8761 ± 0.05	0.9223 ± 0.04
SVM	0.9095 ± 0.02	0.9427 ± 0.02	0.8753 ± 0.02	0.8188 ± 0.02	0.8862 ± 0.02	0.9138 ± 0.03	0.9368 ± 0.02	0.9090 ± 0.03
Naïve Bayes	0.6831 ± 0.03	0.5789 ± 0.01	0.7905 ± 0.03	0.3681 ± 0.04	0.7399 ± 0.05	0.6494 ± 0.05	0.6457 ± 0.03	0.6847 ± 0.02
Decision Tree	0.8641 ± 0.02	0.9234 ± 0.02	0.8031 ± 0.04	0.7277 ± 0.03	0.8284 ± 0.02	0.8734 ± 0.02	0.9105 ± 0.04	0.8632 ± 0.05

The sensitivity was 94.3%, and the specificity was 87.5%, confirming strong classification performance across both groups. The Kappa statistic (0.8188) indicated substantial agreement between predicted and actual classifications. The precision and negative predictive values were 88.6% and 93.7%, respectively, and the F1-score was 0.9138.

**Fig 4:**
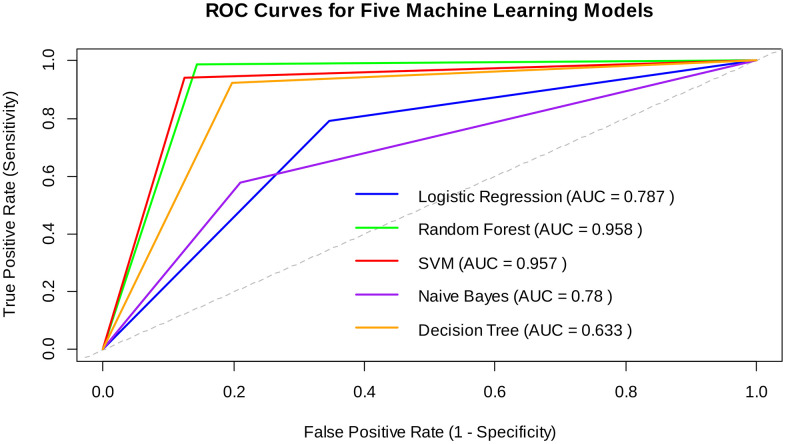
ROC curve analysis of machine learning algorithms with balanced data.

Fig 4 shows that the RF model outperformed all other algorithms, achieving AUC = 0.958, which reflects a strong ability to distinguish between children with and without fever. The SVM model also demonstrated high predictive power, with an AUC of 0.957, indicating similarly outstanding discrimination between fever and non-fever cases.

#### 3.2.3. SHAP value interpretation.

**Fig 5:**
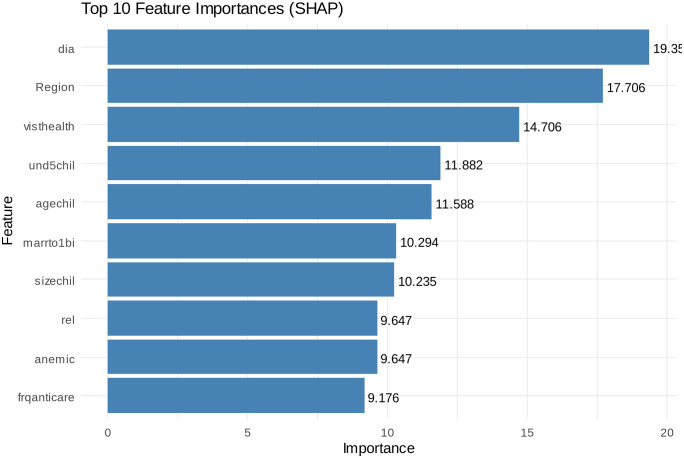
Global feature importance (Mean SHAP Values).

Fig 5 presents a bar plot ranking of the top 10 features based on their mean absolute SHAP values, indicating their average contribution to the model’s predictions across all observations. It provided key insights into the comparative importance of features in the fever prediction model. Diarrhea episodes, region and visiting health facility were identified as the most influential factors of fever status in the model. The top-ranked feature, recent diarrhea episodes, had the highest mean SHAP value of +19.3, indicating a substantial contribution to increasing the log-odds of fever. Assuming the baseline log-odds for fever prediction is 0 (i.e., no feature input), this SHAP value suggests that the presence of diarrhea increases the log-odds of fever to +19.3, translating to a predicted probability of nearly 100%, signifying a dominant influence on the model outcome. The second most important feature, region, showed a mean SHAP value of **+**17.7, highlighting strong geographic differences in fever risk. Similarly, recent visits to a health facility had a SHAP value of **+**14.7, emphasizing that healthcare-seeking behavior is a major determinant of fever status in the model. Other key predictors included: number of under-five children in the household (SHAP value = +11.8), child’s age (SHAP value = +11.3), mother’s age at first birth (SHAP value = +10.3) and size of child at birth (SHAP value = +10.2), had considerable contributions to the model’s predictive capability, each shifting the baseline log-odds of fever significantly. Lower-ranked but still important features like religion and anemia status both had SHAP values of +9.6 and frequency of antenatal care visit had a SHAP value of +9.1, suggesting modest influence on fever prediction.

**Fig 6:**
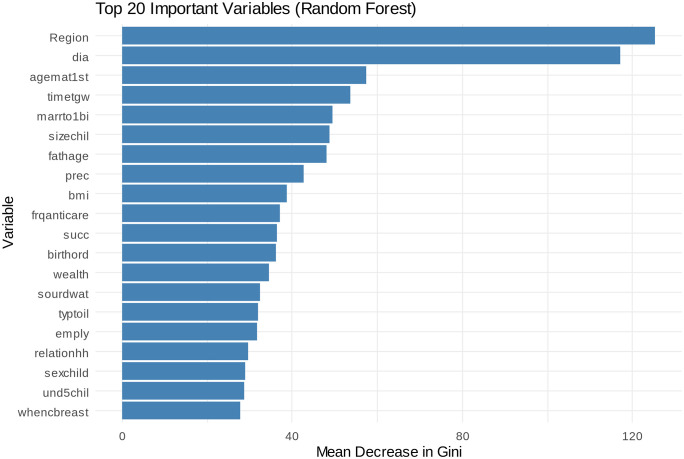
Top 20 most important variables from the RF algorithm based on mean decrease Gini for fever.

Fig 6 displays the top 20 predictors of childhood fever identified by the RF algorithm. The importance of each variable is ranked based on the mean decrease in the Gini index, which measures the contribution of each variable to improving the purity of the classification trees within the ensemble model. Variables with a higher mean decrease in Gini play a more significant role in effectively splitting the data to accurately classify fever cases.

The most influential predictors were region and diarrhea status. Other important predictors included mother’s age at first birth, time taken to access the water source, interval between marriage and first birth, child’s size at birth, father’s age, preceding birth interval, maternal body mass index, frequency of antenatal care visits, wealth index, and type of toilet facility.

The predictors ranked at the lower end of the top 20 list contributed less prominently to the model compared to the highest-ranked variables. These included factors such as relation to the household head, sex of the child, number of under-five children in the household, and the timing of breastfeeding initiation. SHAP values and Gini importance measure feature influence differently. SHAP captures both main and interaction effects on predictions, whereas Gini importance reflects only reduction in node impurity. Consequently, some variables (e.g., visiting a health facility) appeared highly important in SHAP but not rank highly by Gini importance.

#### 3.2.4. Sensitivity analysis.

To address potential data leakage, a sensitivity analysis was conducted by excluding variables that may occur concurrently with or after fever onset, including diarrhea and health facility visits. The ML models were re-trained using the same cross-validation and hyperparameter tuning procedures. The results of the sensitivity analysis indicated that excluding potentially leakage-prone variables resulted in only marginal reductions in predictive performance, suggesting that the models are robust and not substantially influenced by these predictors.

#### 3.2.5. Association rule mining.

The Apriori algorithm was applied to identify patterns associated with fever. A minimum support threshold of 0.05 and minimum confidence of 60% were initially set, balancing the number of rules generated and their reliability. A sensitivity analysis was conducted by varying the support between 0.03 and 0.07 and the confidence between 60% and 90%. The top 10 rules were selected based on a combination of high lift, support, and practical relevance, with actual confidence values ranging from 61.97% to 73.0%. Lift values (4.45–5.24) indicate that these rules represent strong positive associations with fever occurrence [41,42].

**Rule 1**

Children residing in the Tigray region, lacking access to toilet facilities, and who had experienced diarrhea had a 73.0% likelihood of experiencing fever. This pattern was observed among 46 children (support = 0.54%) and had a lift value of 5.24, indicating that these children were more than five times as likely to suffer from fever compared to the overall population.

**Rule 2**

Among children in the Tigray region, those from households without mobile phone ownership and who experienced diarrhea had a 67.1% probability of developing fever. This association applied to 53 children (support = 0.62%) and had a lift of 4.82, indicating a markedly increased risk of fever compared to the general population.

**Rule 3**

Children from the Tigray region, living in households without toilet facilities and who experienced diarrhea, had a 65.8% likelihood of developing fever. This pattern was observed in 50 children (support = 0.58%), with a lift of 4.72, indicating a strong positive association with fever occurrence.

**Rule 4**

Children in the Tigray region, whose mothers were unable to read and who had experienced diarrhea, had a 63.9% probability of developing fever. This association was observed in 46 children (support = 0.54%) and had a lift of 4.59, indicating a strong positive relationship with fever occurrence.

**Rule 5**

Children who were both wasted and underweight, and who had experienced diarrhea, had a 63.2% probability of developing fever. This subgroup comprised 60 children (support = 0.70%) and had a lift of 4.53, highlighting a prevalent and high-risk profile for fever.

**Rule 6**

Children whose mothers had nine or more antenatal care visits, had education below the secondary level, and who experienced diarrhea had a 62.3% probability of developing fever. This pattern was observed in 48 children (support = 0.56%), with a lift of 4.47, indicating a strong association with increased fever risk.

**Rule 7**

Among rural children in the Tigray region who experienced diarrhea, the probability of developing fever was 62.2%. This pattern was observed in 51 children (support = 0.59%) and had a lift of 4.46, indicating a strong association with increased fever risk.

**Rule 8**

Children from households without toilet facilities, who were wasted and had experienced diarrhea, had a 62.2% probability of developing fever. This pattern was identified in 46 children (support = 0.54%) and had a lift of 4.46, indicating a strong positive association with fever risk.

**Rule 9**

Children who were wasted, had experienced diarrhea, and whose caregiver had recently visited a health facility had a 62.2% probability of developing fever. This pattern applied to 46 children (support = 0.54%) and had a lift of 4.46, indicating a strong association with increased fever risk.

**Rule 10**

Children whose mothers had seasonal employment, attended nine or more antenatal care visits, and who experienced diarrhea had a 61.97% probability of developing fever. This subgroup included 44 children (support = 0.51%) and had a lift of 4.45, indicating a strong association with increased fever risk.

## 4. Discussion

This study provides a comprehensive analysis of the factors associated with fever among under-five children in Ethiopia using advanced ML techniques. The findings highlight the potential for developing automated screening tools and clinical decision support systems to aid healthcare providers in the early detection and management of childhood fever.

In this study, five ML algorithms LR, RF, SVM, GNB, and DT were employed to evaluate and compare their predictive performance in forecasting fever among under-five children in Ethiopia. Prior to model training, comprehensive preprocessing steps were carried out, including data cleaning, handling of missing values, target variable balancing, feature engineering, and feature selection. The dataset was partitioned into training and testing subsets using 10-fold cross-validation, and all models were trained on both balanced and unbalanced data. Final predictions were made on unseen test data reserved through the cross-validation process. Evaluation results showed that all five algorithms achieved AUC values above the acceptable threshold, with the RF model outperforming the others. Specifically, the RF achieved an accuracy of 92.2%, AUC of 0.958, sensitivity of 98.7%, specificity of 85.6%, Kappa statistic of 0.8442, positive predictive value of 87.6%, negative predictive value of 92.8%, F1-score of 0.8761, and balanced accuracy of 92.2%. Despite minor variations in performance metrics attributable to differences in socioeconomic context, dataset size, and study settings, the findings align with those reported in prior studies conducted in Ethiopia [22,23], Sub-Saharan Africa [24], South Korea [43], Taiwan [44], and Slovenia [45]. This similarity may be attributed to the nature of the features used across different studies, as the RF algorithm is particularly well-suited for handling categorical variables, high-dimensional data, and complex non-linear relationships, while also requiring relatively minimal hyperparameter tuning [46]. However, our findings contrast with studies conducted on fever of unknown origin [47], as well as those from Brazil [48], Uganda [49], and the USA [50], where RF did not outperform other models. These discrepancies may be attributed to differences in data characteristics, population demographics, evaluation metrics, and potential research biases related to sample size, data preprocessing, and feature engineering methods, all of which can influence model performance and predictive outcomes [51]. Given the superior performance of the RF model we recommend its application in developing data-driven screening tools and clinical decision support systems to identify under-five children at risk of fever. Its high predictive accuracy supports its use in guiding targeted interventions and public health planning, while further validation in diverse settings is encouraged to ensure generalizability.

The bar plot of mean absolute SHAP values in our study revealed that diarrhea status, region, and health facility visits were the top three features of fever among under-five children. These findings align with previous studies in Ethiopia [23,52] and SSA [24]. Diarrhea had the highest SHAP value (+19.3), underscoring the need for early detection and management, along with improved WASH interventions. Region (+17.7) highlighted geographic disparities, calling for targeted, area-specific strategies. Health facility visits (+14.7) emphasized the role of healthcare-seeking behavior, supporting the need to enhance access to and utilization of primary health services. On the other hand, other important predictors that significantly contributed to the model’s predictive performance included the number of under-five children in the household (SHAP value = +11.8), child’s age (+11.3), mother’s age at first birth (+10.3), and size of the child at birth (+10.2). Our findings are consistent with studies conducted in Ethiopia [23,52,53] and SSA [24]. These variables notably influenced the baseline log-odds of fever, underscoring their strong predictive power. Understanding the significance of these features and their impact on the model’s predictions can serve as a basis for evidence-based decision-making, enabling policymakers and public health practitioners to prioritize high-risk groups and design targeted interventions for fever prevention among under-five children.

Our finding showed that lower-ranked but still important features like religion and anemia status both had SHAP values of +9.6 and frequency of antenatal care visit had a SHAP value of +9.1, suggesting modest influence on fever prediction. Our results were consistence with studies in Ethiopia [23,52], SSA [24]. Based on these results, we recommend that public health interventions aimed at reducing fever among under-five children should consider incorporating strategies that address the influence of religion and anemia status, as well as promoting increased frequency of antenatal care visits. Tailoring health education and outreach programs to respect religious contexts can improve community engagement and care-seeking behaviors. Additionally, addressing anemia through nutritional and medical interventions and ensuring pregnant women attend the recommended number of antenatal care visits can modestly but meaningfully contribute to lowering fever risk in children.

In our study, the top ten rules generated by the best-performing model revealed that children residing in the Tigray region, lacking access to toilet facilities, and having experienced diarrhea were most frequently associated with a high probability of fever. This finding is supported by previous studies conducted in Ethiopia [23,54]. The strong association of fever with geographic location, inadequate sanitation, and recent diarrheal illness highlights the critical role of environmental and hygiene-related factors in child health [55,56]. These findings underscore the need for targeted interventions in high-risk regions. We recommend improving sanitation infrastructure, particularly in underserved areas, and integrating diarrhea and fever prevention strategies through strengthened community health programs to reduce the incidence of fever among under-five children.

Children who were both wasted and underweight, and who had experienced diarrhea, were most frequently associated with a high probability of fever. This finding was supported by previous literature Kenya [57]. Based on this finding, we recommend that child health programs prioritize early detection and management of malnutrition, particularly in children who are both wasted and underweight, as they are at higher risk of developing fever. While we are not aware of any machine learning studies using the 2019 EMDHS that directly model fever in under-five children, related work using the same survey provides valuable context. For example, Gebeye et al. (2023) used ML to predict micronutrient deficiency among children using 2019 Mini-DHS data, and identified similar key predictors such as region (especially eastern), poorest wealth, and no maternal education [58]. The findings suggest that underlying socio-demographic determinants are robust across different health outcomes and survey waves, even if direct validation for fever in 2019 is not possible due to data limitations. We used SMOTE to balance the training dataset due to class imbalance. While this improves the model’s ability to detect minority-class (high sensitivity), it may slightly bias evaluation metrics and contribute to the observed lower specificity. Model performance was assessed on the untouched test set to mitigate over fitting, but some inflation of sensitivity relative to specificity cannot be ruled out. Future studies could explore alternative resampling methods or ensemble approaches to further reduce this bias.

## 5. Conclusion

The primary objective of this study was to predict fever among under-five children in Ethiopia using advanced ML algorithms. Additionally, association rule mining through the Apriori algorithm was employed to uncover strong relationships between predictor variables and fever occurrence. Among the five machine learning models evaluated, the RF classifier demonstrated superior predictive performance. Based on these findings, the researcher recommends the development of an AI-based application utilizing the RF algorithm for effective fever prediction in under-five children. Based on SHAP analysis, the most influential predictors of fever among under-five children in Ethiopia were diarrhea status, region, frequency of antenatal care visits, number of under-five children in the household, child’s age, mother’s age at first birth, and child’s size at birth. Similarly, the RF algorithm, using Mean Decrease Gini as a measure of feature importance, identified region, diarrhea status, mother’s age at first birth, time taken to access water, interval between marriage and first birth, child’s size at birth, father’s age, preceding birth interval, maternal body mass index, frequency of antenatal care visits, wealth index, and type of toilet facility as key predictors. Among these, diarrhea status, region, mother’s age at first birth, and child’s size at birth were consistently recognized by both methods, indicating their strong and reliable predictive value for childhood fever. These findings highlight the need for integrated interventions focusing on diarrheal disease prevention, improved maternal and neonatal care, increased ANC utilization, family planning, and region-specific health strategies to effectively reduce the burden of fever in early childhood. Policymakers can use these findings to design targeted, region-specific interventions and strengthen maternal and child health programs. Healthcare providers are guided to prioritize early detection, promote ANC attendance, and deliver integrated care to reduce fever-related morbidity in children.

## 6. Strength and limitations of the study

This study applied five machine learning classification algorithms and association rule mining, enabling robust model comparison and identification of hidden patterns beyond traditional statistical approaches. However, several limitations should be acknowledged. Spatial, social, and macroeconomic variables were not included, which may explain geographic variation in childhood illnesses. The study did not explicitly account for the complex survey design of the EDHS, which may affect representativeness and standard errors. In addition, the use of discrete variables may have influenced model performance, and some predictors (e.g., diarrhea and health service use) may introduce temporal ambiguity and potential data leakage. The analysis is restricted to under-five children in Ethiopia, limiting generalizability to other populations. The study also relied on the 2016 EDHS, which, although the most recent nationally representative dataset with all required variables, may not fully reflect current epidemiological and socioeconomic conditions. Finally, the absence of external validation limits assessment of model generalizability, and reported performance may be optimistic. Future studies should validate these models using independent datasets and incorporate spatial and multi-disease frameworks.

## Abbreviations

ANNs: Artificial neural networks
AUC: Area under the Curve
DT: Decision Tree
EDHS: Ethiopian Demographic and Health Survey
GNB: Gaussian Naïve Bayes
KNN: K-nearest neighbors
LR: Logistic Regression
LMICs: Low- and middle-income countries
ML: Machine learning
EMDHS: Mini Demographic and Health Survey
ROC: Receiver Operating Characteristic
RF: Random Forest
SVM: Support Vector Machine
SMOTE: Synthetic Minority Oversampling Technique
SSA: Sub-Saharan Africa
